# Phase stability and mechanical response of the ordered ScTaCo_2_Sb_2_ crystal: insights from first-principles calculations

**DOI:** 10.1039/d6ra04797a

**Published:** 2026-07-22

**Authors:** Zhang Guo, Shao-Bo Chen, Ai Qin, Chun-Jie Feng, Zhao-Yi Zeng, Xiang-Rong Chen

**Affiliations:** a College of Electronic and Information Engineering, Anshun University Anshun 561000 People's Republic of China shaobochen@yeah.net; b College of Physics and Electronic Engineering, Chongqing Normal University Chongqing 400047 People's Republic of China; c College of Physics, Institute of Atomic and Molecular Physics, Sichuan University Chengdu 610065 People's Republic of China

## Abstract

This study presents a systematic first-principles investigation of the lattice stability, mechanical properties, electronic structure, and lattice thermal conductivity of the Double Half-Heusler compound ScTaCo_2_Sb_2_. The phonon spectrum shows no imaginary frequencies, confirming its lattice dynamic stability. The electronic structure reveals it to be a direct-bandgap semiconductor with a pressure-tunable bandgap. Mechanical calculations confirm that the material satisfies the mechanical stability criteria and exhibits near-critical ductile-to-brittle characteristics. The calculated lattice thermal conductivity is 1.04 W m^−1^ K^−1^, which satisfies the low thermal conductivity requirement for high-efficiency thermoelectric materials. These results demonstrate that ScTaCo_2_Sb_2_ possesses both excellent mechanical stability and low lattice thermal conductivity, positioning it as a promising candidate for thermoelectric applications. While previous work focused on the electronic structure and optical properties of this compound, the present study uniquely addresses its mechanical stability, elastic moduli, ductile-to-brittle transition behaviour, and lattice thermal conductivity, providing essential insights for its potential thermoelectric applications. This work provides a theoretical foundation for the application and materials design of Double Half-Heusler alloys in the field of thermoelectrics.

## Abstract 2

This study presents a systematic first-principles investigation of the lattice stability, mechanical properties, electronic structure, and lattice thermal conductivity of the Double Half-Heusler compound ScTaCo_2_Sb_2_.

## Graphical abstract

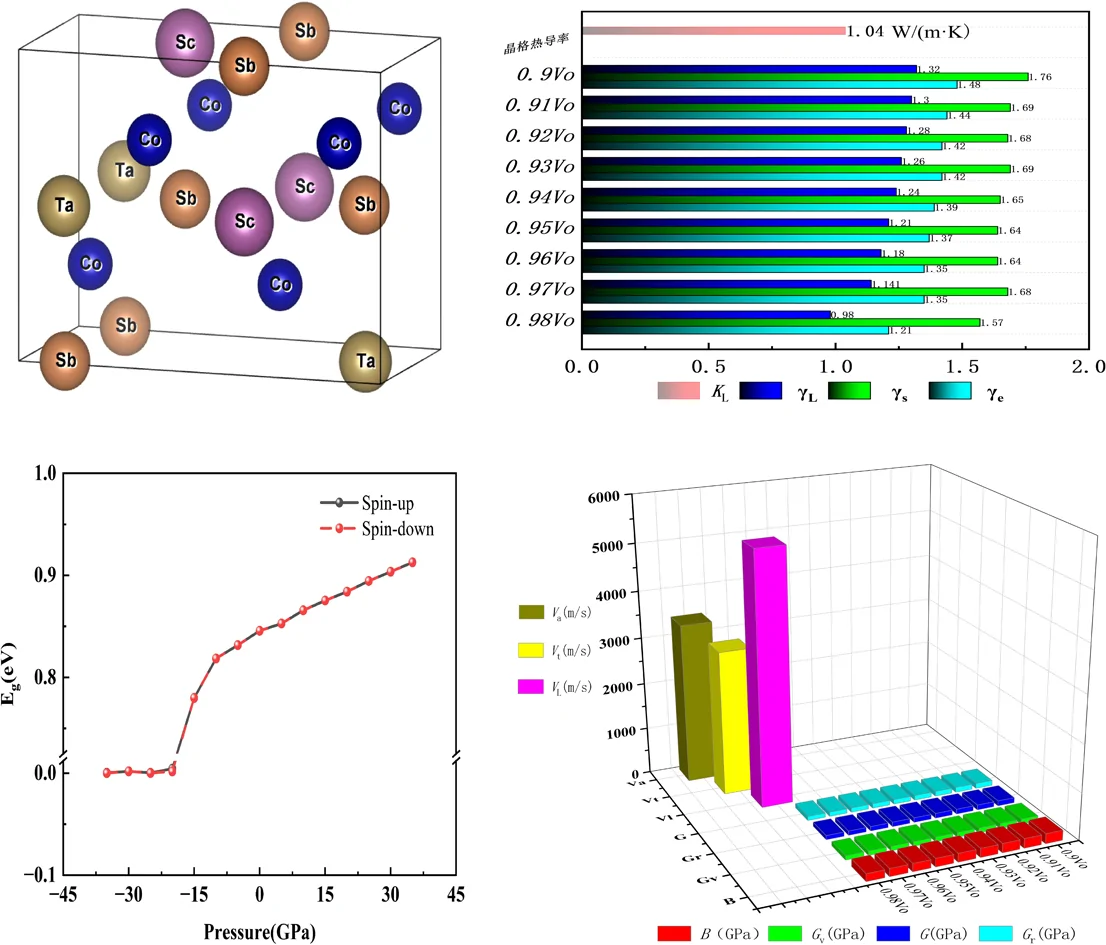

## 1 Introduction

Heusler compounds were discovered by Heusler in 1903 and subsequently named after him.^1^ Owing to the ease of investigation of these through experimental and theoretical calculations, these compounds have become a commonly utilized class of materials in materials science.^2^ The demand for advanced materials is increasingly trending towards multifunctional integration. Over the past several decades, Heusler alloys have become one of the most fascinating and widely investigated material families,^3^ known for their distinctive and adjustable physicochemical characteristics.^4^ They are well-suited for applications in spintronics, optoelectronics, and thermoelectrics.^5–8^ In recent years, numerous Heusler alloys have been explored, and among them, a third class designated as Double Half-Heusler (DHH) alloys has been discovered. These materials are quaternary alloys comprising four distinct atomic species, categorized into three types with the general formulas *X*′*X*″*Y*_2_*Z*_2_, *X*_2_*Y*′*Y*″*Z*_2_, and *X*_2_*Y*_2_*Z*′*Z*″,^9^ respectively. Shashwat Anand *et al.*^10^ investigated these alloys employing identical elemental sets and stability criteria, demonstrating that Double Half-Heusler alloys exhibit suppressed lattice thermal transport, thereby providing a better starting point for optimizing thermoelectric efficiency. As a class of emerging functional materials, Half-Heusler compounds have gradually gained significant attention from the research community. These materials combine excellent magnetic and electronic properties with outstanding structural stability, mechanical strength, and thermal transport performance. Owing to their unique half-metallic characteristics, they exhibit substantial application potential in frontier fields such as spintronics, and have consequently become a research focus in both fundamental science and applied development^11^

The history of Heusler alloys, as reviewed by Dahmane *et al.*,^12^ dates back more than a century to Friedrich Heusler, a German mining engineer, who found that ferromagnetism can be generated by combining Mn and Cu with elements such as Sn, Al, As, Sb, Bi or B, even though none of the constituent elements exhibits intrinsic ferromagnetism.^13^ The demonstration of robust, tunable magnetic order in these intermetallic compounds subsequently triggered an intense wave of investigation, aimed at uncovering the full breadth of their correlated electron physics,^14^ spin-dependent transport phenomena,^15^ and semiconducting thermoelectric behaviour.^16^ To date, theoretical studies on ScTaCo_2_Sb_2_ have been limited to its electronic structure and optical properties. However, its mechanical stability, elastic behaviour, and lattice thermal transport properties-essential for assessing thermoelectric applicability and device reliability-remain unexplored. In this work, we systematically investigate these aspects using first-principles calculations, aiming to fill this gap and provide a comprehensive evaluation of ScTaCo_2_Sb_2_ as a potential thermoelectric material. Specifically, we focus on the mechanical behaviour and lattice thermal transport to comprehensively clarify its thermoelectric potential and the associated fundamental mechanisms.

To the best of our knowledge, only a limited number of studies have evaluated the thermo-mechanical properties of Double Half-Heusler (DHH) alloys, and few publicly available reports have directly focused on ScTaCo_2_Sb_2_. However, as a prerequisite for the practical application of such materials in thermoelectric or spintronic devices, their mechanical properties – which govern stress tolerance and structural integrity during fabrication and service – as well as their intrinsic lattice thermal conductivity, which determines thermoelectric efficiency, have rarely been systematically reported. DHH alloys represent a class of materials that have already established a significant presence across numerous fields. Ongoing theoretical investigations into these alloys and their exhibited properties have rendered them compelling materials. Charifi *et al.*^9^ previously calculated the electronic band structure and optical characteristics of the ScXCo_2_Sb_2_ (X = V, Nb, Ta) alloy series. On this basis, the present work concentrates on the mechanical behaviour and lattice thermal transport of ScTaCo_2_Sb_2_, aiming to comprehensively clarify its thermoelectric potential and the underlying fundamental mechanisms.

## 2 Computational methods

Density functional theory (DFT) was employed as the theoretical basis for this work. First-principles computations were carried out using the Vienna *Ab initio* Simulation Package (VASP).^17^ Electron exchange and correlation were described by the generalized gradient approximation, parametrized by Perdew, Burke, and Ernzerhof (GGA-PBE).^18^ A plane-wave cutoff energy of 500 eV was used, and the Brillouin zone was integrated over a 12 × 6 × 8 *k*-point mesh.^19^ Convergence tests for the plane-wave cutoff energy and *k*-point mesh were performed to ensure the reliability of our computational parameters. The test results are summarized in Table 1, which lists the total energy convergence with respect to different cutoff energies and *k*-point meshes, along with the corresponding optimized lattice constants. The total energy differences between 500 eV and 600 eV for the tested *k*-point meshes are all below 1.1 meV per atom, confirming that a cutoff energy of 500 eV and the 12 × 6 × 8 *k*-point mesh are sufficient to achieve well-converged total energy calculations for ScTaCo_2_Sb_2_. The self-consistent field calculations were converged to an energy tolerance of 10^−6^ eV, while the atomic forces during ionic relaxation were converged to 0.01 eV Å^−1^. The frozen-core approximation was lifted using the projector augmented wave (PAW) formalism to capture the behaviour of valence states in the crystal field of the ions.^20^ To investigate the lattice dynamical stability, phonon spectra were calculated using density functional perturbation theory (DFPT) as implemented in the VASP code. A 2 × 2 × 2 supercell containing 96 atoms was constructed, and atomic displacements of 0.015 Å were applied to compute the force constants. A 6 × 6 × 6 *q*-point mesh was used for integration over the Brillouin zone. The phonon dispersion curves were then interpolated along the high-symmetry path *Γ*–*X*–*M*–*Γ*–*R*–*X* using the Phonopy code.

**Table 1:** Convergence of total energy (in eV) with respect to plane-wave cutoff energy and *k*-point mesh for the Double Half Heusler alloy ScTaCo_2_Sb_2_. The optimized lattice constants (in Å) are also listed for each *k*-point mesh

*k*-point	450 (eV)	500 (eV)	600 (eV)	*a* ≠ *b* ≠ *c* (Å)
8 × 4 × 6	−87.89	−87.89	−87.89	*a* = 4.25 *b* = 8.51 *c* = 6.01
9 × 5 × 7	−87.89	−87.89	−87.89	*a* = 4.25 *b* = 8.51 *c* = 6.01
12 × 6 × 8	−87.89	−87.88	−87.89	*a* = 4.25 *b* = 8.51 *c* = 6.01

## 3 Results and analysis

### 3.1 Crystal structure and phonon stability

**Fig. 1:**
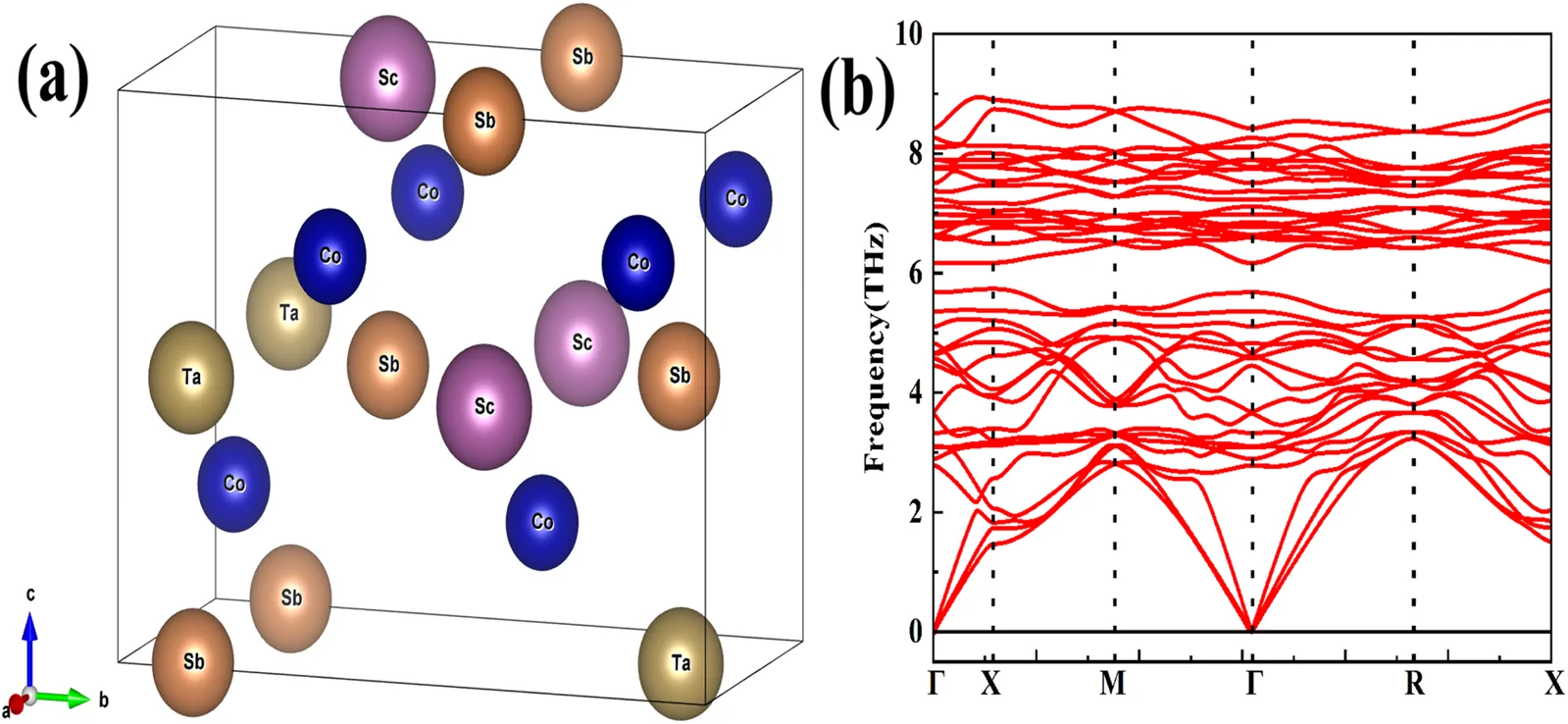
(a) Schematic crystal structure; (b) phonon spectrum of the Double Half-Heusler alloy ScTaCo_2_Sb_2_.

This study focuses on the Double Half-Heusler alloy ScTaCo_2_Sb_2_. The crystal structure of pristine ScTaCo_2_Sb_2_ is shown in Fig. 1(a). The lattice constant obtained in this calculation is *a* = 4.25 Å, *b* = 8.51 Å and *c* = 6.01 Å, in good agreement with the literature value of *a* = 4.244 Å, *b* = 8.506 Å and *c* = 6.014 Å^9^. It adopts an orthorhombic structure with space group *Pmn*2_1_,^9^ which is obtained by combining two Half-Heusler substructures through a specific crystallographic arrangement, with 12 atoms per unit cell. In this structural type, the *X* sites are equally occupied, while the four *Y* sites and four *Z* sites are arranged in a double-tetrahedral coordination. Before evaluating the material's properties, its stability must first be assessed. The phonon dispersion spectrum along the high-symmetry path *Γ* → *X* → *M* → *Γ* → *R* → *X* is shown in Fig. 1(b). Throughout the entire Brillouin zone, no imaginary frequencies are observed in any phonon modes, confirming that this compound possesses good lattice dynamic stability at the equilibrium lattice constant and is free from spontaneous structural distortions. In the phonon spectrum, the acoustic branches exhibit normal dispersive behaviour, while a clear phonon band gap exists between the optical and acoustic branches. No anomalous soft modes are observed, indicating stable interatomic bonding^21^ and regular lattice vibrational behaviour. The optimized geometry thus serves as a reliable prerequisite for probing the material's thermal stability and subsequent investigation of its broader physical behaviour.^22^

### 3.2 Mechanical properties

Having confirmed the structural stability of the alloy, we further evaluate the macroscopic thermo-mechanical properties required for its application as a structural material. Elastic constants^23^ are fundamental for characterizing a material's resistance to deformation. They quantify the deformation behaviour of a material when subjected to applied loads and furnish benchmarks for evaluating its mechanical robustness.^24^

In the field of materials research, assessing a material's resistance to mechanical failure is crucial for creating new compounds with customized functionalities. The Born criteria^25,26^ offer a theoretical basis for evaluating the structural stability of different crystalline systems, thus informing the choice of processing routes and structural regulation during synthesis. The calculated independent elastic constants for ScTaCo_2_Sb_2_ are: *C*_11_ = 247.28 GPa, *C*_12_ = 97.43 GPa, and *C*_44_ = 93.77 GPa. This is essentially consistent with the results calculated by Charifi *et al.* (*C*_11_ = 244.56 GPa, *C*_12_ = 93.78 GPa, and *C*_44_ = 73.50 GPa).^9^ The slight discrepancy in *C*_44_ likely arises from differences in the computational parameters, such as the *k*-point mesh (12 × 6 × 8 in the present work *vs.* 7 × 9 × 5) and the Brillouin-zone integration method (Monkhorst–Pack *vs.* Methfessel–Paxton with a smearing parameter of 0.169 eV). Nevertheless, both sets of constants satisfy the Born stability criteria for the tetragonal structure (as shown below), confirming the mechanical stability of the compound. The derived bulk moduli are also in close agreement (147.38 GPa *vs.* 136.87 GPa), indicating that the overall mechanical behavior predicted by the two studies is qualitatively consistent. For a half-metallic alloy with a tetragonal lattice belonging to the *X*′*X*″*Y*_2_*Z*_2_ family, the tetragonal mechanical stability requirements listed below must be fulfilled:^26^

1 *C*_11_ > 0, *C*_44_ > 0, *C*_11_ + 2*C*_12_ > 0, *C*_11_ − *C*_12_ > 0

Inserting the computed values into the Born stability conditions demonstrates that the elastic constants of the ScTaCo_2_Sb_2_ alloy meet all the Born criteria, confirming that the material's inherent configuration is mechanically stable. Following the Voigt–Reuss–Hill scheme,^27–29^ the volume modulus *B* and the shear modulus *G*^29^ are obtained from the computed data as follows:2
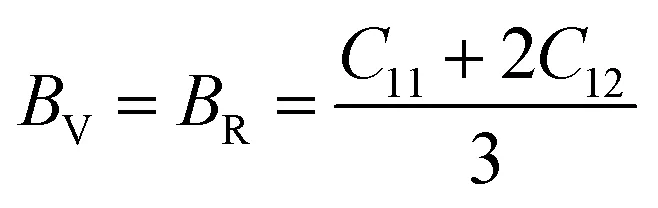
3
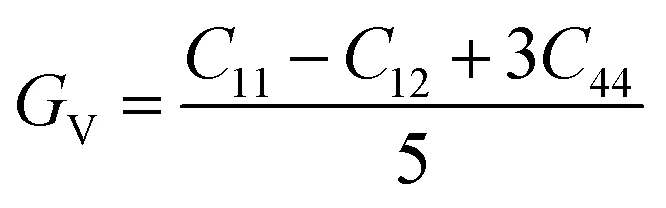
4
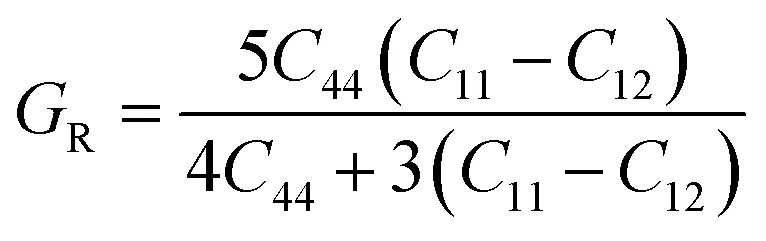


In the set of calculated quantities—specifically *B*_V_, *B*_R_, *G*_V_ and *G*_R_—the subscripts “V” and “R” denote the Voigt and Reuss schemes, respectively, which serve as the two fundamental constituents of the Voigt–Reuss–Hill averaging approach. The Hill model is subsequently constructed by arithmetically averaging the results derived from the Voigt and Reuss formulations:^30^5
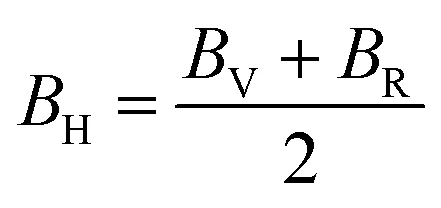
6
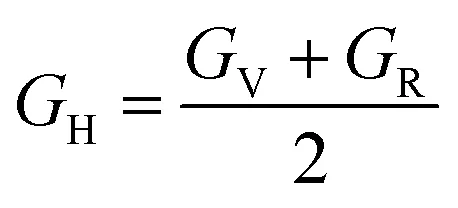


Using well-established theoretical relationships, *E* can be obtained from *B* and *G* as follows:^31^7
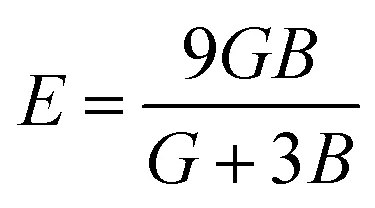
8
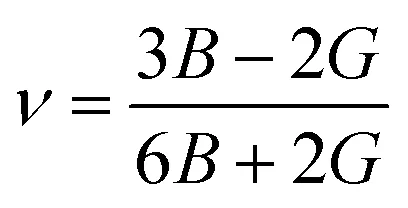


Hardness^32,33^ is a key parameter for characterizing the hardness of a material. The Vickers hardness is calculated using the following formula:^32^

9 *H*_v_ = 0.92 * *k*^1.137^ * *G*^0.708^

in the formula *k* = *G*/*B*.

To distinguish ductile from brittle behaviour in a material, the Cauchy pressure (*C*_p_) is a commonly invoked criterion mainly used to assess that tendency, notably when evaluating the mechanical response of intermetallic compounds or complex alloys. The Cauchy pressure can be obtained from the elastic constants *via* a standard formula*C*_p_ = *C*_12_ − *C*_44_.^34^ Inserting the calculated *C*_12_ and *C*_44_ values for this alloy into the formula yields a positive Cauchy pressure. This indicates enhanced ductility, which favours plastic deformation (dislocation slip) over brittle fracture.

Based on DFT computations, the intrinsic elastic moduli and related physical characteristics of the ScTaCo_2_Sb_2_ Heusler alloy have been obtained. These computed results are presented in Table 2. The volume modulus (*B*), together with the shear modulus (*G*), extracted from the elastic constants, serves as a key parameter for evaluating a material's ability to withstand volumetric and resist shear deformation. The *B*/*G* ratio is used to empirically assess ductility or brittleness. As proposed by Pugh, a *B*/*G* value exceeding 1.75 typically suggests ductile behaviour, while a lower value indicates brittleness. The *B*/*G* ratio of this Double Half-Heusler ScTaCo_2_Sb_2_ alloy is 1.72, which lies slightly below the critical threshold of 1.75, indicating a tendency towards brittleness according to this criterion. Notably, the positive Cauchy pressure (*C*_p_ = 3.66 GPa) generally implies dominant metallic bonding and a tendency towards ductility. This competing behaviour between ductile and brittle criteria suggests that the alloy resides in the critical region of the ductile-to-brittle transition, exhibiting near-critical ductile-to-brittle characteristics.^35^ To further clarify this interpretation, we note that the *B*/*G* ratio reflects the overall resistance to plastic flow and fracture, while the Cauchy pressure is more sensitive to the directional nature of bonding and the angular character of interatomic forces. Such mixed indicators are not uncommon in complex intermetallics and suggest that ScTaCo_2_Sb_2_ lies near the intrinsic ductile–brittle boundary. Additionally, Poisson's ratio is 0.26, which is below the empirical brittle/ductile threshold of 0.33, further supporting the tendency toward brittleness. The positive Cauchy pressure (3.66 GPa) suggests the presence of some metallic bonding character, which may contribute to moderate ductility. The combination of these complementary criteria indicates that the alloy exhibits near-critical ductile-to-brittle characteristics.

**Table 2:** The calculated volume modulus *B*, shear modulus *G*, Young's modulus *E*, Cauchy pressure *C*_p_, *B*/*G* ratio, Poisson's ratio, and Vickers hardness *H*_v_ of uncompressed ScTaCo_2_Sb_2_

Crystal structure types	*B* (GPa)	*G* (GPa)	*E* (GPa)	*C*_p_ (GPa)	*B*/*G*	*ν*	*H*_ν_ (GPa)
ScTaCo_2_Sb_2_	147.38	85.20	215.39	3.66	1.72	0.26	11.56
	136.87 (ref. 9)	81.24 (ref. 9)	203.47 (ref. 9)	—	—	—	—

**Fig. 2:**
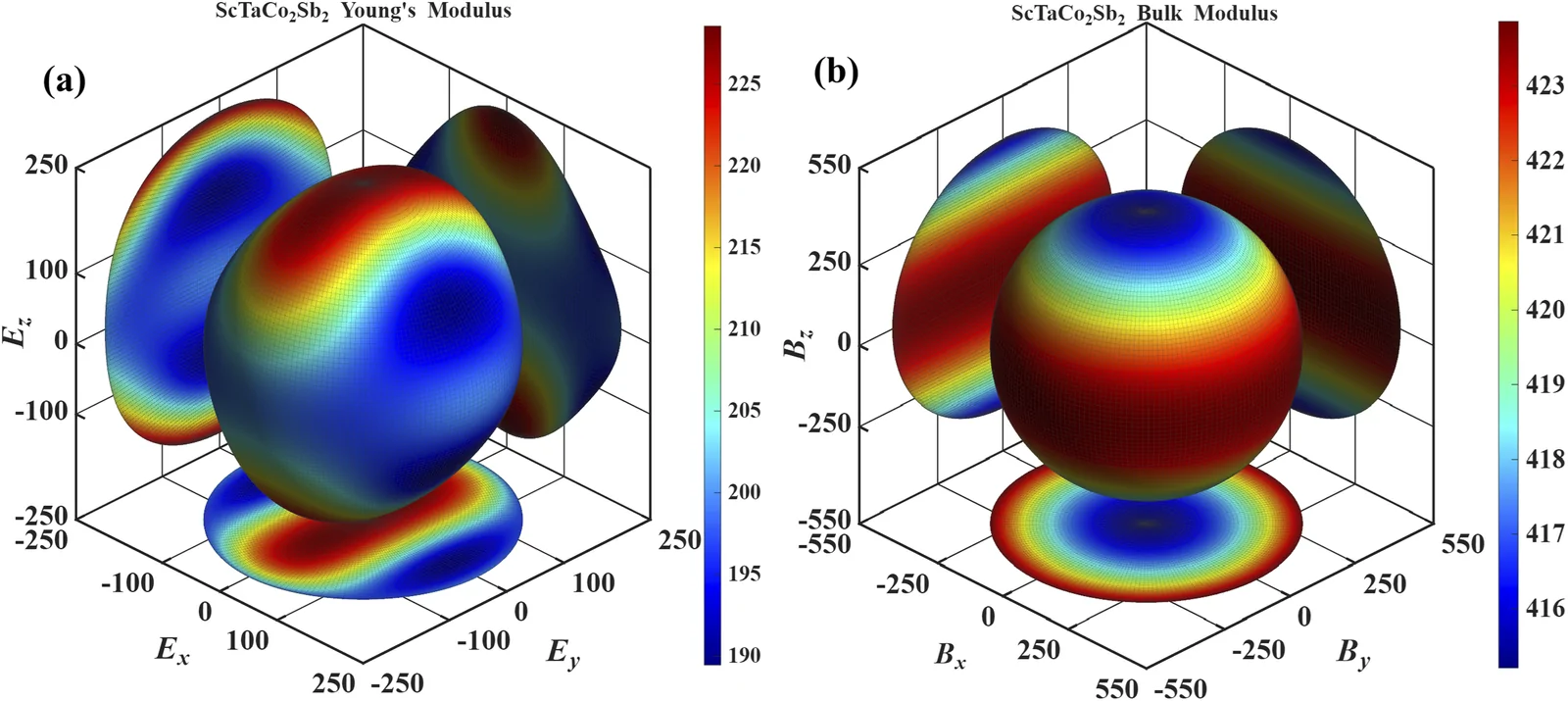
3D Young's modulus and bulk modulus of the Double Half-Heusler alloy; (a) 3D contour of the bulk modulus; (b) 3D contour of Young's modulus.

Both Young's modulus and the shear modulus are core indicators for evaluating the mechanical properties of materials. Fig. 2(a) and (b) present three-dimensional projections of the volume modulus and Young's modulus, respectively. As shown in the figures, this Double Half-Heusler alloy ScTaCo_2_Sb_2_ exhibits high stiffness, a high volume modulus, and a certain degree of mechanical anisotropy, which are generally consistent with the data presented in Table 2. The high volume modulus and Young's modulus indicate that these materials can maintain good structural integrity when used as structural components or subjected to stress environments. Furthermore, a thorough understanding of the anisotropy of Young's modulus is crucial for the future design of microelectronic or functional devices based on these materials, as device performance may be influenced by grain orientation and the direction of the applied stress field. This exceptional mechanical stability, together with the previously discussed thermodynamic stability and half-metallic electronic characteristics, collectively establishes the application potential of this class of Half-Heusler alloys in advanced spintronic devices.

### 3.3 Electronic structure

**Fig. 3:**
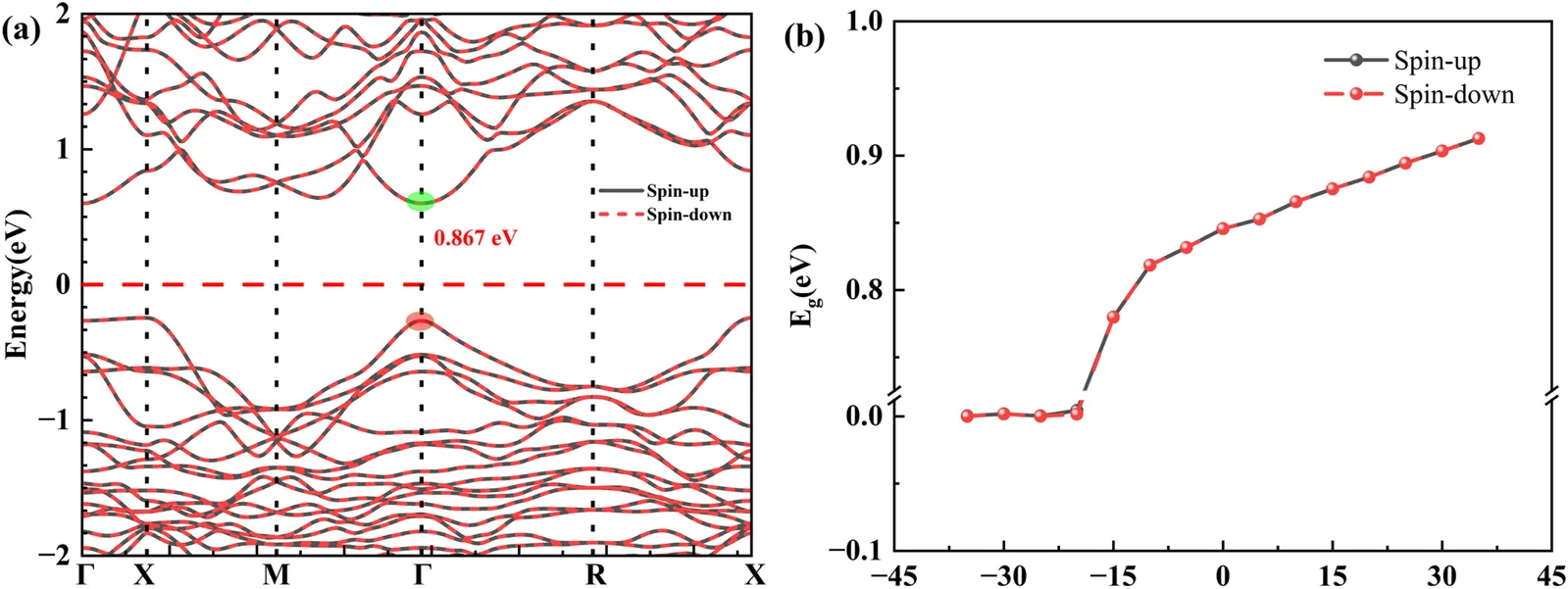
(a) Band structure of the Double Half-Heusler alloy ScTaCo_2_Sb_2_; (b) pressure-dependent bandgap behaviour of the alloy.

The electronic band structure of ScTaCo_2_Sb_2_ was investigated using first-principles calculations, as shown in Fig. 3(a). At the equilibrium lattice constant, the compound behaves as a non-magnetic direct-bandgap semiconductor, with both the valence band maximum and the conduction band minimum located at the centre of the Brillouin zone (the *Γ* point), exhibiting a bandgap of 0.867 eV. The spin-up and spin-down bands almost completely overlap, and no noticeable spin splitting is observed, indicating that the ground state of the system is spin-degenerate, lacking half-metallic character or significant spin polarisation. Near the *Γ* point, the valence and conduction bands exhibit relatively flat dispersions, suggesting large effective masses for the band-edge carriers and consequently relatively low carrier mobility.

To examine the effect of pressure on the electronic configuration of the system, we calculated the bandgap (*E*_g_) variations for the spin-up and spin-down channels under varying pressure conditions, with the results shown in Fig. 3(b). As the positive (compressive) pressure increases, the band gaps of both spin channels gradually widen, indicating that compression enhances the band gap of the system. In the negative pressure (tensile) regime, the bandgap decreases with increasing tension, eventually approaching zero, whereupon the system exhibits metallic conduction behaviour. Overall, pressure can induce a continuous transition from metallic to semiconducting behaviour in this system, with a monotonic increase of the bandgap with pressure, demonstrating that the material responds favourably to pressure tuning of its electronic structure. The pressure-induced bandgap variation originates from changes in the p–d hybridization between the Co-d and Sb-p states. Under compression, the reduced interatomic distances enhance the orbital overlap, leading to stronger hybridization and a wider bandgap. Under tension, the increased bond lengths weaken the p–d hybridization, resulting in bandgap narrowing. When the tensile strain reaches −25 GPa, the bandgap closes completely, leading to a semiconductor-to-metal transition. This interpretation is further supported by the band structure analysis at selected pressures, which shows systematic shifts in the valence band maximum and conduction band minimum positions under strain.^36,37^

**Fig. 4:**
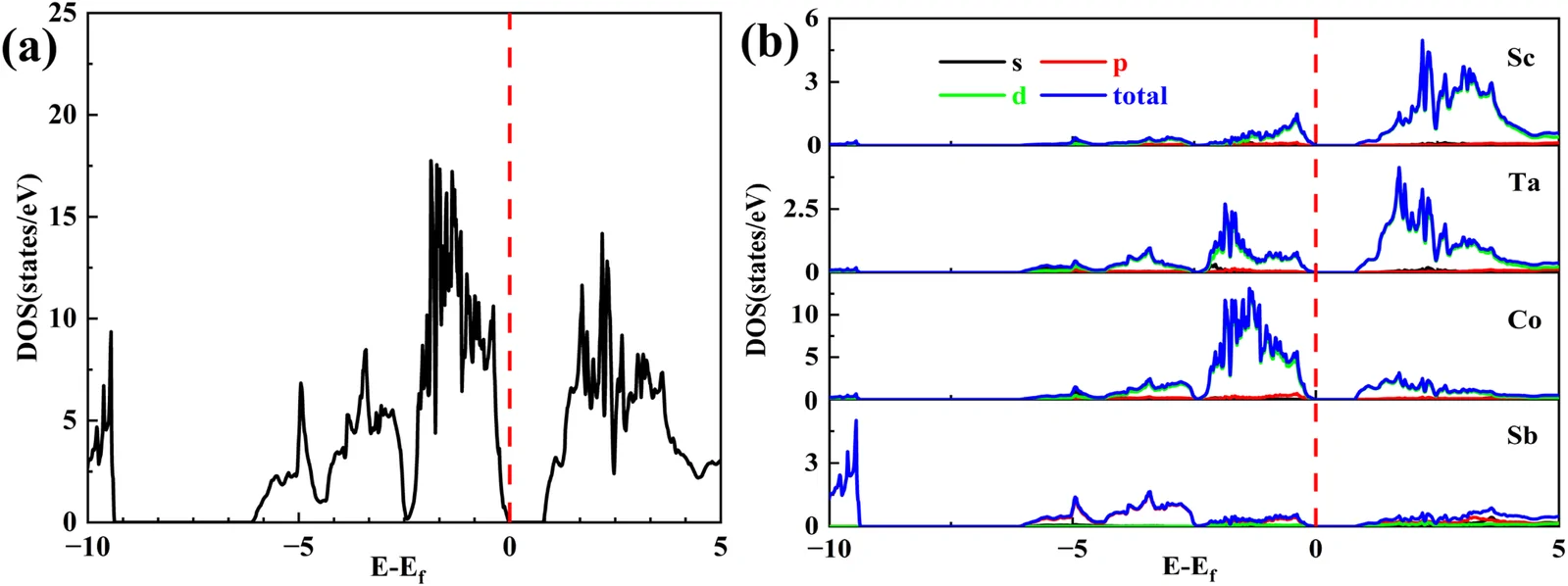
Partial density of states of the Double Half-Heusler alloy: (a) total partial density of states; (b) element-resolved partial density of states.

Differences in macroscopic mechanical properties originate fundamentally from the microscopic electronic structure and chemical bonding. To gain deeper insight into the observed mechanical behaviour, we calculated and analyzed the electronic density of states (DOS) of the system. The variations in mechanical properties primarily arise from the influence of different elemental compositions on the electronic structure and interatomic bonding characteristics.^38–40^ The DOS of the alloy is presented in Fig. 4, which verifies the electronic structure calculations. The origin of the differences in macroscopic mechanical behaviour lies in the distinct electronic structures and bonding characteristics resulting from compositional variations. The DOS analysis reveals that all systems possess a non-zero density of states at the Fermi level, consistent with semiconducting behaviour. Based on the DOS and projected density of states (PDOS) analysis, a qualitative prediction of the macroscopic mechanical characteristics of the Double Half-Heusler alloy ScTaCo_2_Sb_2_ can be made: owing to strong metal–covalent hybridized bonding dominated by Co-d orbitals, the alloy exhibits a high bulk modulus, high elastic modulus, and high intrinsic hardness, indicating excellent strength and stiffness. However, the d-orbital-dominated DOS distribution near the Fermi level also suggests a pronounced brittle tendency and limited ductility.^41^ Moreover, the continuity and uniformity of the DOS distribution indicate high structural stability and weak mechanical anisotropy, enabling a stable mechanical response under conventional loading conditions. Hence, this alloy is suitable for application as a structural-functional material characterized by high strength and low plasticity.

**Fig. 5:**
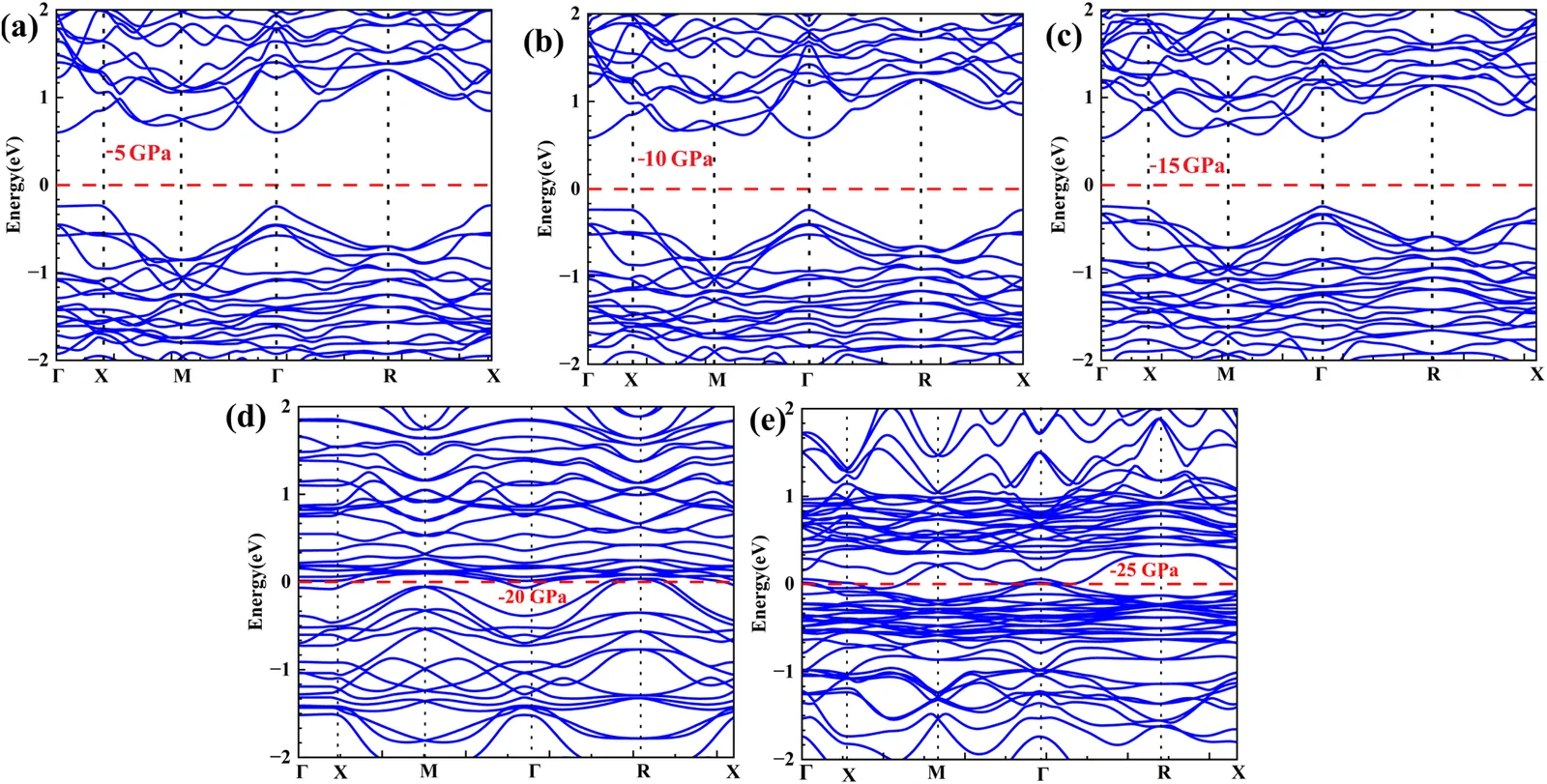
Bandgap behaviour under applied tensile stress: (a) −5 GPa, (b) −10 GPa, (c) −15 GPa, (d) −20 GPa, and (e) −25 GPa.

To further elucidate the electronic configuration under various pressures, the band structure was computed for pressures spanning from −5 GPa to −25 GPa, with the results shown in Fig. 5(a)–(e). At lower pressures (−5 GPa to −15 GPa), no bands cross the Fermi level, and a clear energy gap exists between the valence band maximum and the conduction band minimum, indicating that the material behaves as a semiconductor in this pressure regime. As the negative pressure increases from −5 GPa to −15 GPa, the bandgap gradually narrows, exhibiting a pressure-induced bandgap narrowing effect. When the pressure reaches −25 GPa, bands cross the Fermi level, and the bandgap completely disappears, marking the transition to a metallic state. Moreover, the bands become denser and flatter overall, suggesting a significant increase in the electronic density of states under high pressure – a trend that is fully consistent with the previously observed pressure dependence of the bandgap.

### 3.4 Thermodynamic properties

The energy conversion efficiency of thermoelectric materials is largely governed by their lattice thermal conductivity, which is a critical thermoelectric parameter.^42^ Because acoustic modes play a crucial role in heat transfer in semiconductors, Slack^43^ proposed a method for deriving an expression for the lattice thermal conductivity under specific temperature conditions:10
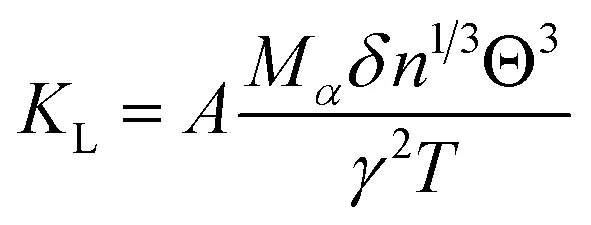
where *A* is11
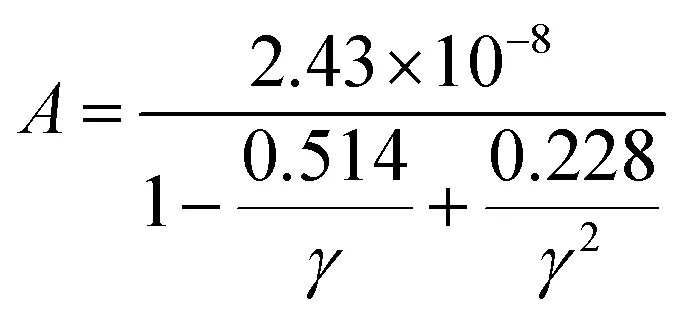
Here, *M*, *δ*^3^, *n*, *γ*, and *Θ* denote the mean atomic mass, the atomic volume, the atom count in the elementary cell, the sound-mode Grüneisen parameter, and the sound-mode Debye temperature, respectively. This formula has been widely adopted for estimating phonon thermal conductivity. The Debye temperature associated with the acoustic branches and the Grüneisen parameters can be accurately determined from the phonon dispersion relation, with the relevant dispersion data obtainable either from lattice dynamics calculations or experimental measurements. The Grüneisen parameter *γ*_ω_ characterizes the strength of anharmonic effects in the crystal lattice. Its determination requires quasi-harmonic phonon calculations or relies on experimentally measured thermal expansion coefficients. In the absence of these conditions, Xiao *et al.*^44^ proposed a simplified formula that estimates the Grüneisen parameters using variables such as elastic properties, Poisson's ratio, or sound velocity.

Following the method of Jia *et al.*,^42^ we rapidly estimate the Grüneisen parameters and lattice thermal conductivity based on elastic parameters (bulk modulus, shear modulus, and sound velocity) obtained from first-principles calculations (Tables 3 and 4).

**Table 3:** Lists the bulk modulus *B*, the Voigt and Reuss shear moduli *G*_V_ and *G*_r_, their average *G* (the shear modulus), the long-wavelength *γ*^e^_L_, the shear *γ*^e^_s_, and the average acoustic Grüneisen parameter *γ*_e_ of compressed ScTaCo_2_Sb_2_ (calculated relevant values referring to *V*_0_, Table 2)

Compressed volume	*B* (GPa)	*G*_v_ (GPa)	*G*_r_ (GPa)	*G* (GPa)	*γ* ^e^_L_	*γ* ^e^_s_	*γ*_e_
0.98*V*_0_	160.30	90.36	88.96	89.66	0.98	1.57	1.21
0.97*V*_0_	167.41	93.14	91.74	92.44	1.41	1.68	1.35
0.96*V*_0_	172.76	95.73	94.23	94.98	1.18	1.64	1.35
0.95*V*_0_	179.02	98.34	96.70	97.52	1.21	1.64	1.37
0.94*V*_0_	185.01	101.06	99.31	100.18	1.24	1.65	1.39
0.93*V*_0_	192.60	103.82	101.92	102.87	1.26	1.69	1.42
0.92*V*_0_	198.14	106.56	104.44	105.50	1.28	1.68	1.42
0.91*V*_0_	204.86	109.50	107.20	108.35	1.30	1.69	1.44
0.90*V*_0_	214.32	112.48	109.98	111.23	1.32	1.76	1.48

**Table 4:** The longitudinal sound velocity *V*_L_, transverse sound velocity *V*_S_, average sound velocity *V*_a_, acoustic Grüneisen parameter *γ*_e_, the value of *A*, average atomic mass *M*, acoustic Debye temperature *Θ*_e_, the cube root of the atomic volume *δ*^3^, and lattice thermal conductivity *K*_L_ of the ScTaCo_2_Sb_2_ alloy

Alloy category	*V*_L_ (m s^−1^)	*V*_S_ (m s^−1^)	*V*_a_ (m s^−1^)	*γ*_e_	*M* (g mol^−1^)	*Θ*_e_ (K)	*δ* ^3^	*K*_L_ (K)
ScTaCo_2_Sb_2_	5375.94	3076.87	3418.49	1.35	48.94	93.42	2.02	1.04
	5516 (ref. 47)	2727 (ref. 47)	3070 (ref. 47)					<1 (ref. 48)
	5226 (ref. 9)	3008 (ref. 9)	3340 (ref. 9)					

Grüneisen parameters: *γ*_e_. The Grüneisen parameter *γ*_i_ describes the relationship between a phonon frequency *ω*_i_ and the volume change *V*, and is defined as follows:^45^12
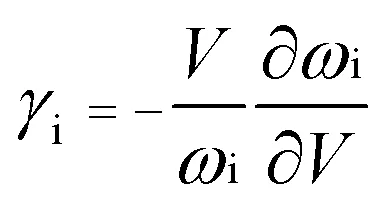


In the long-wavelength limit, the phonon frequency *ω*_i_ is proportional to the sound velocity: *ω*_i_ = *v*_i_*q*, where *q* is the wave vector. Substituting *ω*_i_, the longitudinal sound velocity (eqn (15)), and the shear sound velocity (eqn (16)) into eqn (11), we can derive the equations for the long-wavelength γ^L^_e_, the shear *γ*^s^_e_, and the average acoustic Grüneisen parameter *γ*_e_:^42^13
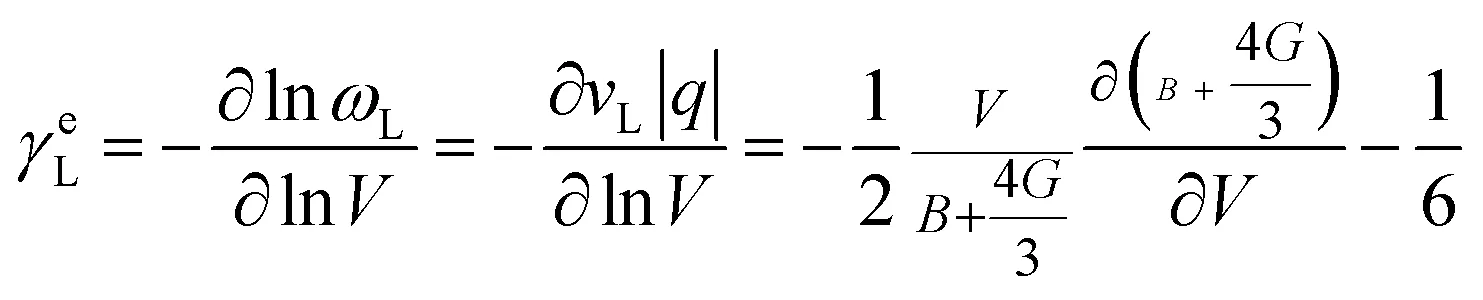
14
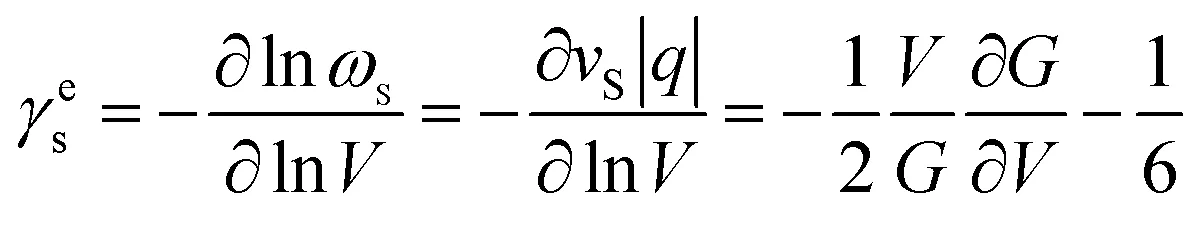
15
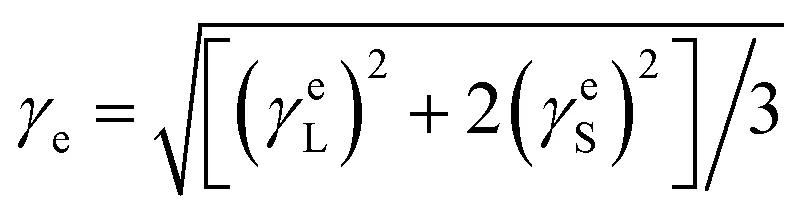


The longitudinal (*V*_L_) and transverse (*V*_S_) sound velocities, together with the associated mean velocity *V*_a_, are given by the following expressions.:^46^16
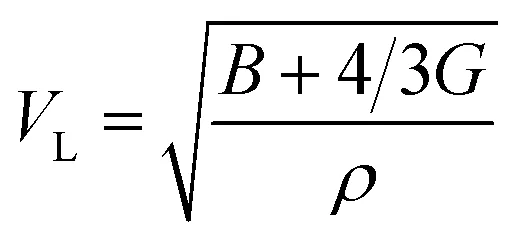
17
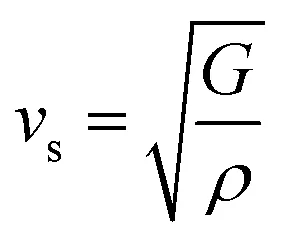
18
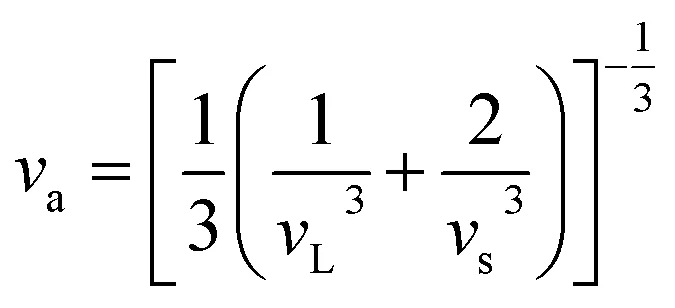
where *ρ* denotes the density of the material. Furthermore, the acoustic Debye temperature can be written as a function of the sound velocity as follows:^42^19
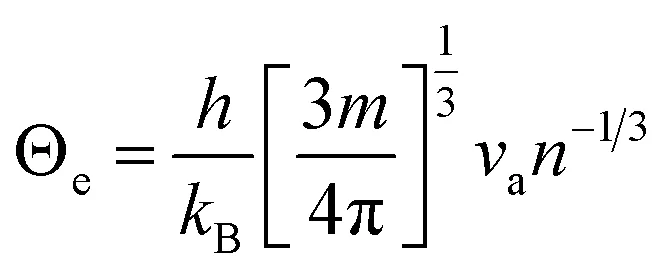
Here, *h* denotes Planck's constant, *k*_B_ is Boltzmann's constant, and *m* is the atomic number density. The acoustic Debye temperature derived from the elastic properties characterizes the overall vibrational spectral features.

Through our calculations, we systematically obtained key thermal transport parameters of the ScTaCo_2_Sb_2_ alloy, including sound velocities, the acoustic Grüneisen parameter, the acoustic Debye temperature, and the lattice thermal conductivity. The calculated longitudinal sound velocity *V*_L_ is 5375.94 m s^−1^, and the transverse sound velocity *V*_S_ is 3076.87 m s^−1^, which are consistent with the sound velocity characteristics of covalent/metallic bonding, reflecting strong interatomic interactions in the material. The acoustic Grüneisen parameter *γ*_e_ is 1.35, indicating significant phonon anharmonicity in this alloy, which provides a physical basis for the generation of low lattice thermal conductivity. The anisotropy parameter *A* is 3.1 × 10^−6^, suggesting that the mechanical and thermal transport characteristics of this material exhibit weak anisotropy and are nearly isotropic overall. Meanwhile, the calculated acoustic Debye temperature *Θ*_e_ is 93.42 K; the relatively low acoustic Debye temperature corresponds to low phonon vibrational frequencies and low phonon group velocities, further corroborating the intrinsically low thermal conductivity of the material. The resulting lattice thermal conductivity *K*_L_ is 1.04 W m^−1^ K^−1^, which lies in the low range and satisfies the core requirement for low lattice thermal conductivity in high-performance thermoelectric materials.

## 4 Conclusion

Using DFT calculations, this work carries out a detailed first-principles examination of the ground-state geometry, elastic stability, electronic states, mechanical response, and lattice thermal transport of the Double Half-Heusler compound ScTaCo_2_Sb_2_. The computed phonon dispersion shows no imaginary branch throughout the whole Brillouin zone, verifying its robust dynamical stability. Electronic structure analysis shows that ScTaCo_2_Sb_2_ behaves as a direct-bandgap semiconductor featuring an equilibrium bandgap of 0.867 eV. Mechanical property calculations demonstrate that this alloy satisfies the mechanical stability criteria for tetragonal crystal systems, with a volume modulus *B* of 147.38 GPa, shear modulus *G* of 85.20 GPa, and Young's modulus *E* of 215.39 GPa. The *B*/*G* ratio is 1.72, approaching the critical value of the Pugh criterion for the ductile-to-brittle transition, exhibiting near-critical ductile-to-brittle characteristics. The positive Cauchy pressure (3.66 GPa) further supports its moderate ductility. Regarding thermal transport properties, the lattice thermal conductivity *K*_L_, derived from the Slack model based on elastic parameters, is 1.04 W m^−1^ K^−1^, which is relatively low. The acoustic Grüneisen parameter *γ*_e_ is 1.35, indicating moderate anharmonicity in the system, which is favourable for reducing lattice thermal conductivity. The acoustic Debye temperature *Θ*_e_ is 93.42 K, a relatively low value that further corroborates the intrinsically low thermal conductivity of this material. In summary, ScTaCo_2_Sb_2_ possesses good mechanical stability, near-critical ductile-to-brittle behaviour, and low lattice thermal conductivity, positioning it as a strong candidate for thermoelectric applications. This work provides a theoretical basis for performance optimization and thermal transport regulation in Double Half-Heusler alloy systems for thermoelectric applications.

## Author contributions

Zhang Guo: writing – review & editing, supervision, investigation. Shao-Bo Chen: writing – review & editing, supervision, conceptualization. Ai Qin: writing – review & editing, writing – original draft, investigation, formal analysis, data curation, conceptualization. Chun Jie Feng: writing – review & editing, investigation, formal analysis. Zhao-Yi Zeng: writing – review & editing, investigation, software. Xiang-Rong Chen: writing – review & editing, supervision, investigation, conceptualization.

## Conflicts of interest

There are no conflicts to declare.

## Data Availability

All data that support the findings of this study are included in the article.
